# Vaccination with synthetic long peptide formulated with CpG in an oil-in-water emulsion induces robust E7-specific CD8 T cell responses and TC-1 tumor eradication

**DOI:** 10.1186/s12885-019-5725-y

**Published:** 2019-06-06

**Authors:** Sean K. Maynard, Jason D. Marshall, Randall S. MacGill, Li Yu, Jennifer A. Cann, Lily I. Cheng, Michael P. McCarthy, Corinne Cayatte, Scott H. Robbins

**Affiliations:** 1 Microbial Sciences, MedImmune, Gaithersburg, MD USA; 2 Statistical Sciences, MedImmune, Gaithersburg, MD USA; 3 Translational Sciences (Pathology), MedImmune, Gaithersburg, MD USA

**Keywords:** Cancer vaccines, Synthetic long peptides (SLP), Toll-like receptor 9 (TLR9), Human papilloma virus antigen E7 (HPV E7)

## Abstract

**Background:**

Despite considerable efforts at developing therapeutic vaccines for cancer, clinical translation of preclinical successes has been challenging, largely due to the difficulty of inducing strong and sustained cytotoxic T lymphocyte (CTL) responses in patients. Several peptide-based cancer vaccines have failed to show sustainable tumor regression in the clinic, possibly because of a lack of optimization of both the adjuvant and antigen components of the preparations. Here, we aimed to develop and optimize a vaccine format utilizing a synthetic long peptide (SLP) containing the human papilloma virus 16 (HPV16) E7 antigen, with a centrally located defined MHC class I epitope, and evaluate its immunogenicity and efficacy in combination with various adjuvant formulations.

**Methods:**

E7_31–73_ SLP was tested alone or in combination with toll-like receptor (TLR)3, TLR4, TLR7/8 and TLR9 agonists and formulated in oil-in-water (o/w) or water-in-oil (w/o) emulsions to determine a vaccine format inducing a robust CD8 T cell response in murine models. Once a lead vaccine format was determined, we examined its ability to inhibit tumor growth in the murine TC-1 model that expresses HPV16 E7 antigen.

**Results:**

We identified the TLR9 agonist CpG formulated in a squalene-based o/w emulsion as the most potent adjuvant, inducing the expansion of multifunctional antigen specific CD8 T cells with cytolytic potential. We also demonstrated that SLP E7_31–73_ + CpG + o/w emulsion vaccine can provide prophylactic and more importantly, therapeutic benefit in the TC-1 murine tumor model.

**Conclusions:**

Our results demonstrate that the novel vaccine format E7 SLP + CpG delivered in an o/w emulsion holds potential for the promotion of strong CTL responses and tumor eradication and encourages further development of peptide/adjuvant vaccines in cancer immunotherapy strategies.

**Electronic supplementary material:**

The online version of this article (10.1186/s12885-019-5725-y) contains supplementary material, which is available to authorized users.

## Background

The generation of a durable CD8 T cell response with strong cytolytic function specific to a diverse array of tumor antigen epitopes is the most important immune objective of cancer vaccines. Preclinical development in cancer vaccines has explored several formats that have been proven effective for such an immune outcome. This included DNA plasmid vaccination, dendritic cell (DC)-targeting molecules, whole cell vaccines derived from tumor cells or antigen-loaded DCs, stabilized RNA-based vaccines, and virally-vectored constructs derived from vaccinia, poxvirus, and adenovirus (reviewed in [1, 2]). Peptide-based cancer vaccines have also been under development for many years and vaccination with peptides alone can, at times, show some efficacy and limited tumor regression in pre-clinical models [3]. However, long-lasting suppression of tumor growth has generally been shown to require the formulation of peptide-based vaccines with immune-enhancing adjuvants (reviewed in [4]).

Several potent adjuvants have been developed from the class of compounds known as toll-like receptor (TLR) agonists, which mimic pathogen-associated molecular patterns (PAMPs) or conserved pathogen-derived compounds, to activate the host innate immune response. Adjuvant comparison studies have identified synthetic TLR agonists that trigger TLR3, TLR7, and TLR9 as promising adjuvant candidates for cancer vaccines as they mimic the RNA and DNA-based PAMPs dominant in viral challenges, which often lead to strong cytolytic CD8 T cell responses, critical for effective antitumor immunotherapy [5–7]. TLR9 short oligodeoxynucleotide (ODN) agonists are most conducive for formulation as they are easy to manufacture, exhibit strong potency in humans [8, 9], and do not require special formulations to protect from degradation [10, 11].

Several adjuvanted peptide vaccines have been tested clinically and although peptide-specific CD8 T cell responses can be detected at low levels, substantial clinical efficacy has been limited [12, 13]. These results may be due to suboptimal formulations using short peptides which lack antigen presenting cell (APC)-activating properties and adjuvants (e.g. Montanide) that are not engineered for favorable MHC class I cross-presentation or improved homing to the draining lymph node [14]. Here, we report that pairing CpG delivered in an o/w emulsion with a 43 amino acid Synthetic Long Peptide (SLP) derived from HPV16 E7 resulted in vigorous CD8 T cell responses characterized by cytokine multifunctionality, establishment of memory populations, and high cytolytic potential. This vaccine format was also curative in a TC-1 tumor model through day 120 and was protective against tumor re-challenge.

## Methods

### SLP synthesis

SLPs for HPV16 E7 43 amino acid in length were designed so the immunodominant H-2D^b^-restricted epitope (RAHYNIVTF) was centrally located. HPV16 E7_31–73_ SLP sequence: SSEEEDEIDGPAGQAEPDRAHYNIVTFCCKCDSTLRLCVQSTH. SLPs were synthesized to > 90% purity by New England Peptide and determined to have endotoxin levels < 0.2 EU/mg by Endosafe endotoxin reader (Charles River).

### Vaccines

Peptides (3–20 μg) were delivered in PBS pH 7.2 (Gibco) or in formulation with AddaVax (InvivoGen) or Montanide ISA720 (Seppic) which comprised 50% v:v of the 100 μl dose administered per mouse. Addavax formulations vaccine components were mixed by vortexing for 20 s, and Montanide formulations were mixed using two syringes linked by a 3-way luer lock valve. Adjuvant doses were selected based on recommended range by manufacturer for use in mice. Adjuvants tested were CpG ODN 2395, poly(I:C), R848 (all InvivoGen) and MPLA (Avanti Polar Lipids).

### Immunogenicity studies

Vaccinations were delivered to C57BL/6 J female mice between 6 and 10 weeks of age (Jackson Labs) by subcutaneous (s.c.) injection at the ventral base of the tail on days 0 and 21. Mice were housed in the MedImmune Laboratory Animal Resource facility using microisolator cages using Teklad corncob bedding (Envigo) using a standard 12 h light/dark cycle. All mice were euthanized by CO_2_ and spleens were harvested. Spleens were processed by GentleMACS (Miltenyi Biotec), washed with 5% FBS-RPMI-1640, and homogenates were centrifugated at 300 x g for 10 min at 4 °C, before performing RBC lysis with ACK Lysing Buffer (Invitrogen) for 2 min at room temperature. All animal studies were performed under the review and approval of the MedImmune Institutional Animal Care and Use Committee (IACUC).

### Flow cytometric assays

Flow cytometry staining was carried out on freshly prepared single cell splenocytes preparations. Splenocytes were cultured for 5 h with 1 μg/mL E7_49–57_ peptide in the presence of 1:1000 GolgiPlug (BD Biosciences). Cells were fixed with BD CytoFix and permeabilized as needed with BD Perm/Wash buffer. Antibodies used for FACS analyses included CD8α (53–6.7), CD62L (MEL-14) CD4 (GK1.5), CD44 (IM7), KLRG1 (2F1), IL-2 (JES6-5H4), IFN-γ (XMG1.2), TNF-α (MP6-XT22); B220 (RA3-6B2), (BD Biosciences); HPV16 E7_49–57_ Dextramer (Immudex). All cell populations were gated on single live cells. For detection of multifunctional CD8 T cells, splenocytes were gated for B220^−^ > CD8^+^ cytokine^+^. For detection of T effector memory (T_EM_) cells, splenocytes were gated for B220^−^ > CD8^+^ CD44^hi^ dextramer^+^ > CD62L^−^ KLRG ^hi^.

### In vivo cytotoxicity assay

Splenocytes from naïve C57BL/6 J mice were harvested, counted, and divided equally into two groups for labeling as target populations with carboxyfluorescein succinimidyl ester (CFSE) (Sigma) at either 10 μM (CFSE^hi^) or 1 μM (CFSE^lo^). Additionally, splenocytes were washed and pulsed with either 1 μg/mL HPV16-E7 peptide pool (CFSE^hi^) or an irrelevant EBV-gp100 peptide pool (CFSE^lo^) for 1 h at 37 °C. Peptide pools were 15-mers overlapping by 11 covering the entire sequences of E7 and gp350 and synthesized by Anaspec. Both target cell populations were mixed together at a 1:1 ratio (confirmed by FACS), and 2 × 10^8^ combined target cells in 200 μL were injected by tail vein injection into vaccinated mice. 4 h after cell transfer, recipient mice were sacrificed and spleens harvested. Single cell splenocytes were analyzed by FACS for presence of CFSE-positive target cells. Percent E7-specific killing was calculated by the following formula (1 – (CFSE^hi^ / CFSE^lo^)) × 100.

### ELISpot

The ELISpot assay was performed as described in [15], using the mouse IFN-γ ELISpot kit (BD Biosciences) per manufacturer’s instructions. Briefly, 96-well plates were coated with anti-mouse IFN-γ antibody overnight at 4 °C. Splenocytes were stimulated at 5e5 cells/well with 1 μg/mL E7_49–57_ for 24 h at 37 °C. Plates were washed and incubated with biotinylated anti-mouse IFN-γ detection antibody and then streptavidin-HRP. Development of signal occurred by exposure to AEC substrate, then scanning and analysis performed on the AID vSpot Spectrum (Autoimmun Diagnostika).

### TC-1 tumor model

All animal experiments were conducted in accordance with guidelines established by the MedImmune Institutional Animal Care and Use Committee. The TC-1 tumor line, syngeneic to C57BL/6 mice, was obtained from American Type Culture Collection (Cat # CRL 2785), and maintained in DMEM + 5% FBS + 1% penicillin/streptomycin (all Gibco). Sub-confluent cultured cells were harvested in 10 mM EDTA, washed, and resuspended in DMEM + 10 mM HEPES (Gibco) for injection. 2e4 viable TC-1 cells in a volume of 30 μL were implanted subcutaneously into the left hind footpad. Mice were randomly assigned to treatment or control groups at this point. Tumor growth was evaluated by direct measurement with calipers, beginning at day 10. Bi-directional measurements were collected every 2–4 days, and tumor volume calculated using the formula: volume = (length x width^2^)/2 [16]. Mice were euthanized when the primary tumor exceeded 1000 mm^3^ in accordance with ethical guidelines. For vaccination, 3–20 μg E7 SLP was formulated with 20 μg CpG ODN 2395 in AddaVax and PBS in a total volume of 100 μL. Vaccinations were administered s.c. into the ventral surface of the base of tail. At various time points after vaccination, spleens and/or tumor tissue were harvested from sacrificed mice (selections were randomized when subsets of vaccinated groups were euthanized), processed through GentleMACS, and analyzed for immune cell subsets by FACS. In some cases, mice were re-challenged with TC-1 tumor and implanted with 2e4, 5e4, or 1e5 TC-1 cells in the right footpad on day 120.

### Histopathology and immunohistochemistry

Select tumor samples were collected via blunt dissection of footpad tissue, immersion-fixed in 10% neutral buffered formalin for 12–24 h, processed routinely, and embedded in paraffin. Briefly, 4 μm sections were collected on Plus-charged slides, and immunohistochemistry was performed on a Dako Autostainer-Plus Slide Stainer using an anti-Mouse CD8α (Clone 4SM15, eBioscience) at 1:5000 dilution. Detection was performed using goat anti-rat (Jackson Immuno) at 2 μg/mL, followed by Vectastain Elite ABC HRP Standard Peroxidase Kit (Vector Laboratories), visualized with ImmPACT DAB Peroxidase (Vector Laboratories), and counterstained with Gills #2 Hematoxylin (Richard-Allen Scientific). Rat IgG2aĸ (BD Pharmingen) at 1:5000 served as isotype control. Rat spleen was used as the positive control tissue. All samples were examined by a board-certified pathologist for detection of CD8^+^ cells within the tumor center.

### Statistical analysis

The one-way ANOVA model with heterogeneous within group variance was used for comparisons between groups and *p*-values were adjusted for multiple comparisons. The log-rank test is used for comparison of survival curves. SAS 9.3 was used for the analysis.

## Results

### Combining synthetic long peptide with CpG induces an expansion of antigen specific CD8^+^ T cells

**Fig. 1:**
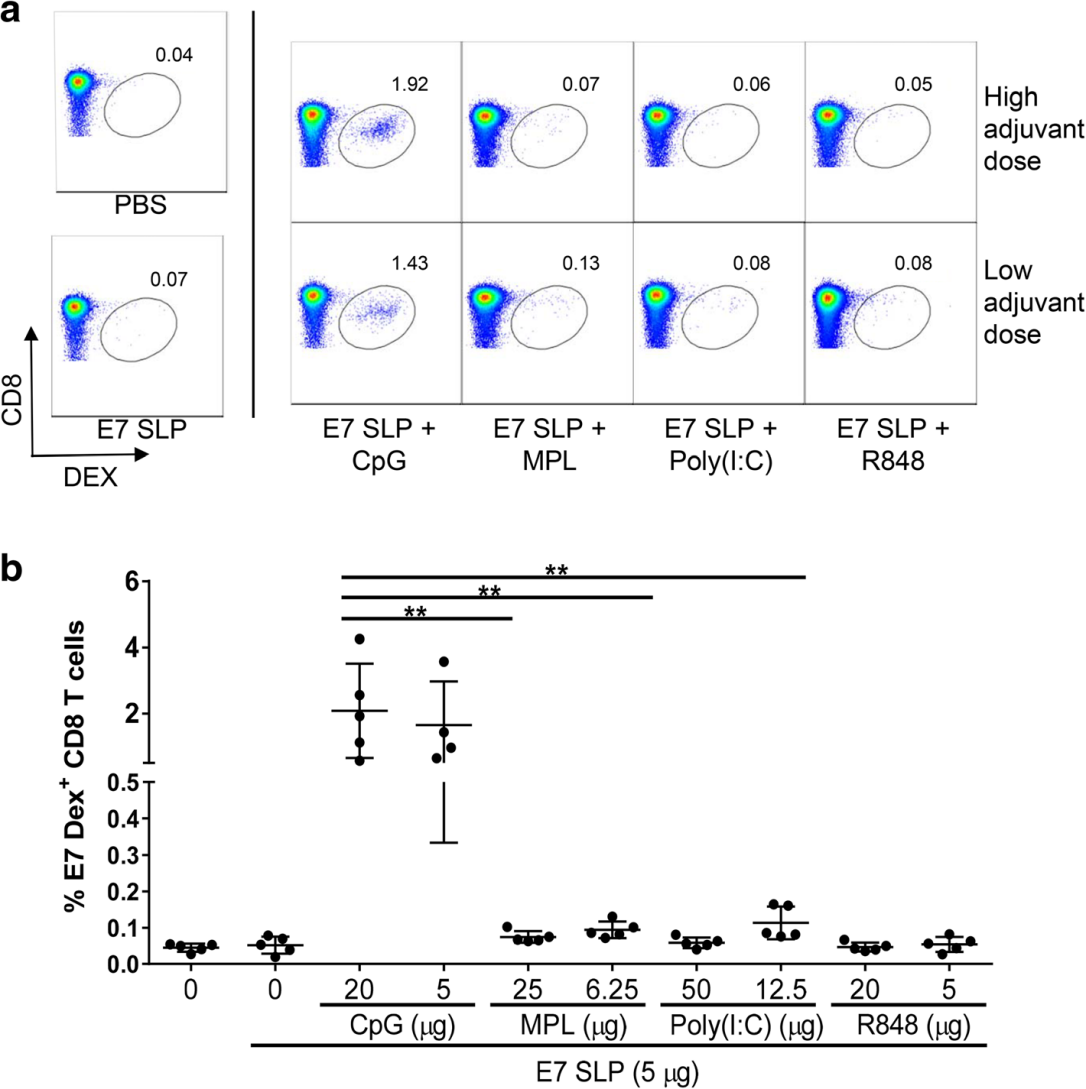
E7 SLP adjuvanted with CpG promotes antigen specific CD8 T cells expansion. Mice (*n* = 5) were immunized s.c. on day 0 and 21 with 5 μg E7 SLP adjuvanted with the TLR agonists and indicated doses. Splenocytes were isolated on day 28 and stained with E7-specific dextramers for FACS analysis. **a** Representative dot plots of E7 antigen specific dextramer staining of CD8 T cells. **b** The percentage of E7-specific dextramer^+^ CD8 T cells is presented for each vaccine condition. Two-sided hypothesis test for comparisons using one-way ANOVA model **, *p* < 0.01

The generation of a robust and durable CTL response with strong cytolytic function against tumors is a critical objective for a successful cancer vaccine. To achieve this, we developed a HPV16 E7 SLP which incorporated a centrally located H-2D^b^ CD8 epitope RAHNIVTF and tested it in combination with either the TLR9L CpG, TLR4L MPL, TLR3L Poly(I:C) or TLR7/8 L R848 to determine the most potent TLR agonist to induce expansion of antigen specific CD8 T cells. Mice were immunized s.c. (base of tail) on days 0 and 21 with E7 SLP alone or E7 SLP plus either a dose of TLRL commonly used in murine vaccine studies or a four-fold lower dose of TLRL and expansion of antigen specific CD8 T cells was measured by dextramer staining in the spleens on day 28. Figure 1a shows representative dot plots of E7 dextramer staining and Fig. 1b illustrates that expansion of E7 antigen specific CD8 T cells was optimally promoted by the TLR9L CpG and superior to expansion seen with any of the other TLRLs tested.

### SLP combined with CpG and formulated with an oil-in-water emulsion is a superior adjuvant for the induction of antigen specific CD8 T cells

While the results from Fig. 1 show an expansion of antigen specific CD8 T cells when E7 SLP is paired with CpG, a successful therapeutic cancer vaccine likely would need to generate a more robust immune response. While there are many strategies currently investigated to enhance the immunogenicity of vaccines, we explored the use of the two basic types of emulsions, the water-in-oil (w/o) and the oil-in-water (o/w) to deliver our SLP + TLR agonists to enhance vaccine CD8 T cell responses.

**Fig. 2:**
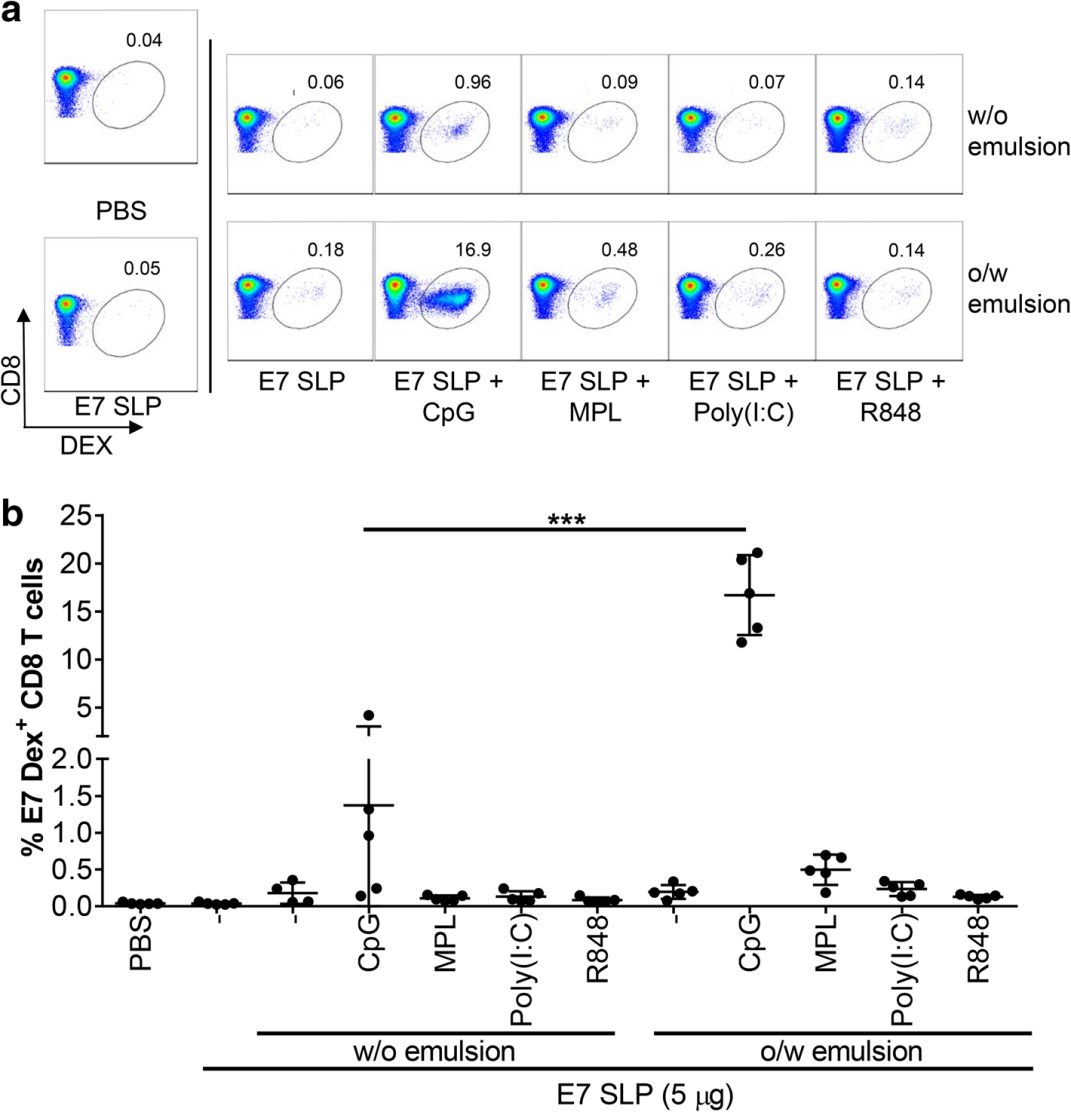
E7 SLP + CpG + o/w emulsion superior induction of antigen specific CD8 T cells. Mice (*n* = 5) were immunized s.c. on day 0 and 21 with 5 μg E7 SLP +/− 20 μg CpG, 25 μg MPL, 50 μg poly(I:C), or 20 μg R848, and formulated in either w/o emulsion or o/w emulsion. Splenocytes were isolated on day 28 and stained with E7-specific dextramer for FACS analysis. **a** Representative dot plots of E7-specific dextramer staining of CD8 T cells. **b** The percentage of E7-specific dextramer^+^ CD8 T cells is presented for each vaccine condition. Two-sided hypothesis test for comparisons using one-way ANOVA model ***, *p* < 0.001

To determine the optimal pairing of TLRL, SLP and emulsion that generate a robust expansion of antigen specific CD8 T cells, mice were vaccinated on days 0 and 21 with E7 SLP combined with either TLR9L CpG, TLR4L MPL, TLR3L Poly(I:C) or TLR7/8 L R848 and formulated with either w/o or o/w emulsions and spleens were harvested on day 28 and stained with E7 dextramer. As shown in Fig. 2 E7 SLP + CpG + o/w emulsion resulted in a greater than 10-fold expansion in E7 antigen specific CD8 T cells when compared to the response observed with E7 SLP + CpG + w/o emulsion. The E7-specific CD8 T cell response observed with CpG + o/w emulsion was also significantly higher than that observed with the other TLR-agonist formulated in either the w/o or o/w emulsions.

### SLP + CpG + o/w emulsion induces strong cytotoxic T cell responses that demonstrates in vivo killing and establishment of long lasting responses

**Fig. 3:**
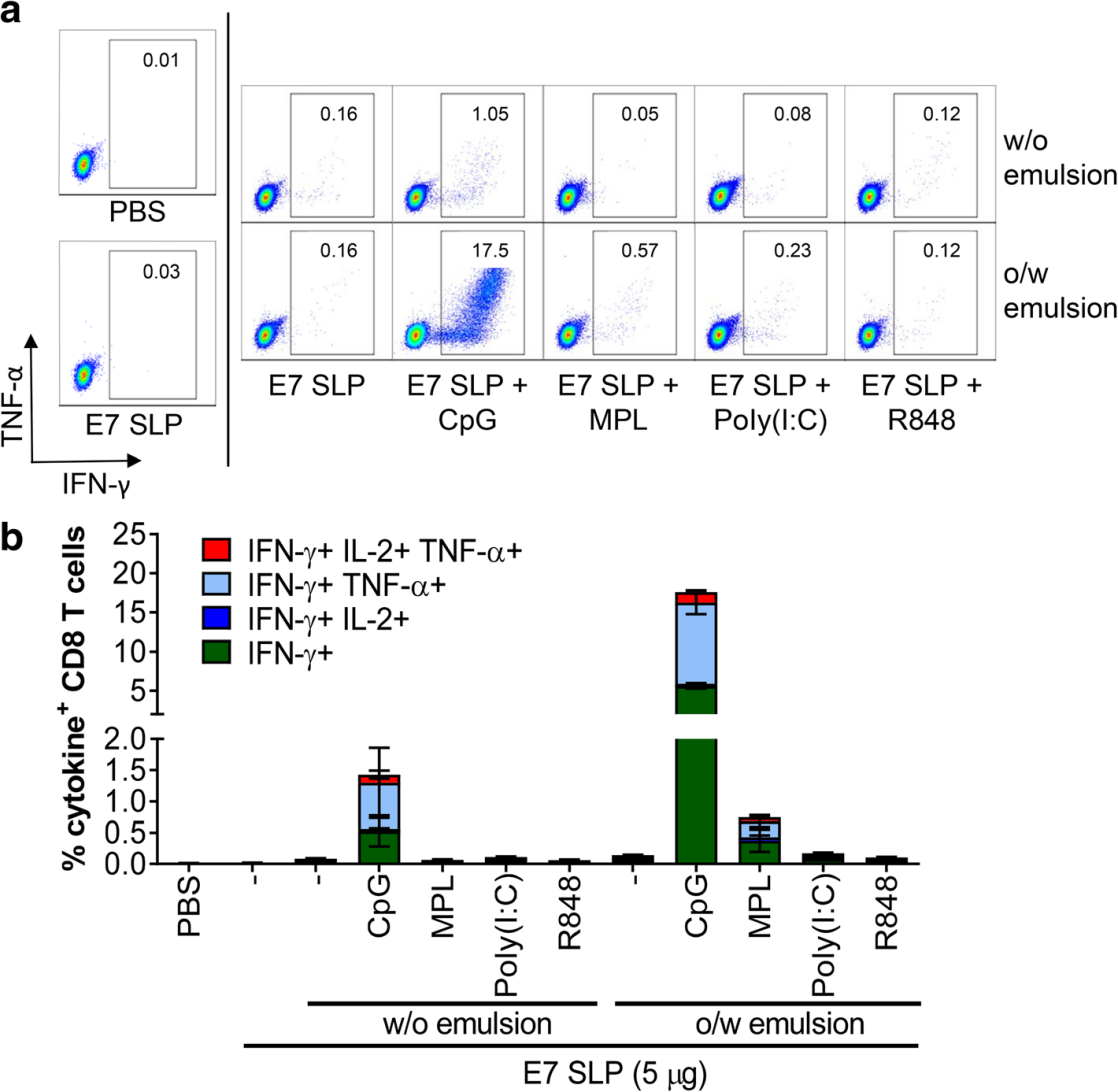
E7 SLP + CpG + o/w emulsion induces a robust CD8 T cell expansion. Mice (n = 5) were immunized s.c. on day 0 and 21 with 5 μg E7 SLP adjuvanted with 20 μg CpG, 25 μg MPL, 50 μg poly(I:C), or 20 μg R848, and formulated in either w/o emulsion or o/w emulsion. Splenocytes were isolated on day 28. Cells were restimulated with E7 ME peptide for 5 h, then FACS analyzed for E7-specific multifunctional CD8 T cells. **a** Representative dot plots for intracellular expression of TNF-α and IFN-γ by CD8 T cells is presented **b** Individual polyfunctionality profiles with percentages of intracellular TNF-α, IL-2, and IFN-γ are presented for each condition

CD8 T cells responsible for clearing tumors in tumor-bearing mice are characterized by high cytolytic capability and long-lasting durability. To determine the quality of CD8 T cell responses achieved by vaccination with SLP + CpG + o/w or w/o emulsion, we performed an ex vivo stimulation with the E7 peptide RAHNIVTF on splenocytes harvested from the groups of animals previously described in Fig. 2. Representative dot plots of these analyses illustrate the robust polyfunctional TNF-α and IFN-γ production induced by ex vivo stimulation in the E7 SLP + CpG + o/w emulsion groups (Fig. 3a) and the percent of IFN-γ producing CD8 T cells found in the spleens of vaccinated animals clearly demonstrating the superiority of SLP + CpG + o/w emulsion over all other test conditions (Fig. 3b).

**Fig. 4:**
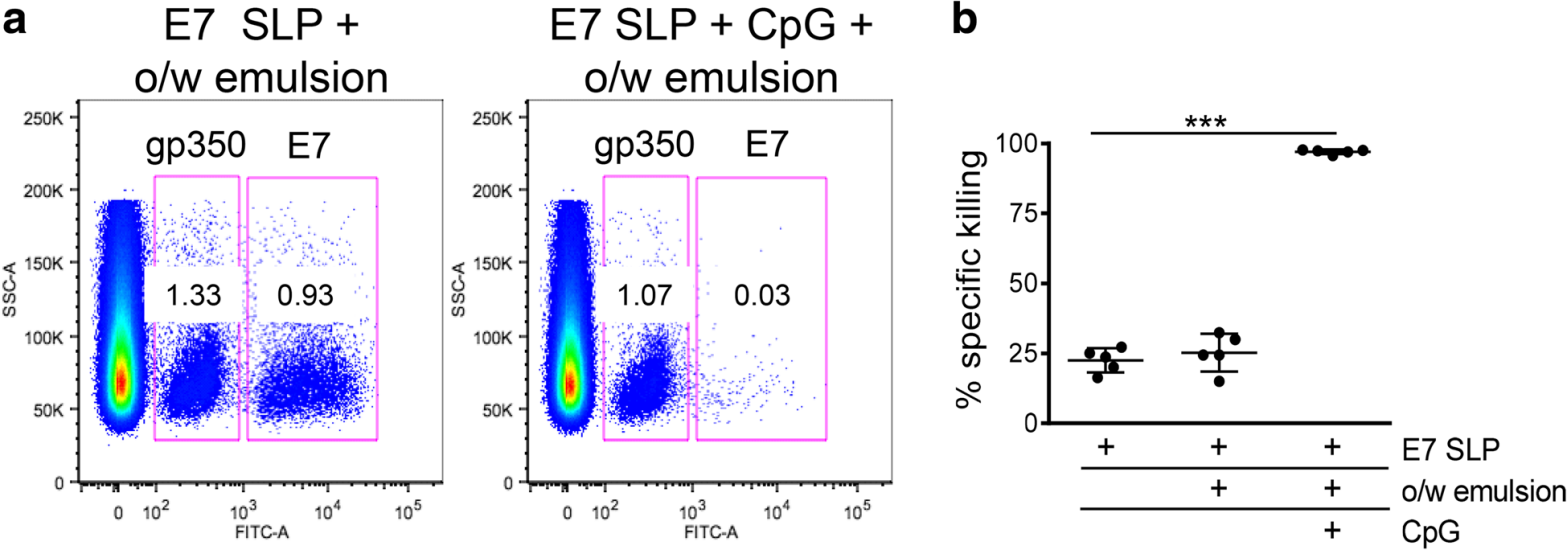
Adjuvanted E7 SLP promotes robust E7-specific cytotoxic responses in vivo. a Mice (n = 5) were immunized s.c. on days 0 and 21 with 20 μg E7 SLP +/− CpG +/− emulsion. Two populations of naïve syngeneic splenocytes were labeled with 1 and 10 μM CFSE and loaded with irrelevant EBV-gp350 peptide pool and E7 peptide pool, respectively. After 1:1 ratio mixing, splenocytes were injected i.v. into vaccinated mice on day 28. Splenocytes were harvested 4 h later and FACS analyzed for CFSE content. **b** Percentages of E7-specific killing for each vaccine condition was evaluated. Two-sided hypothesis test for comparisons using one-way ANOVA model; ***, *p* < 0.001

**Fig. 5:**
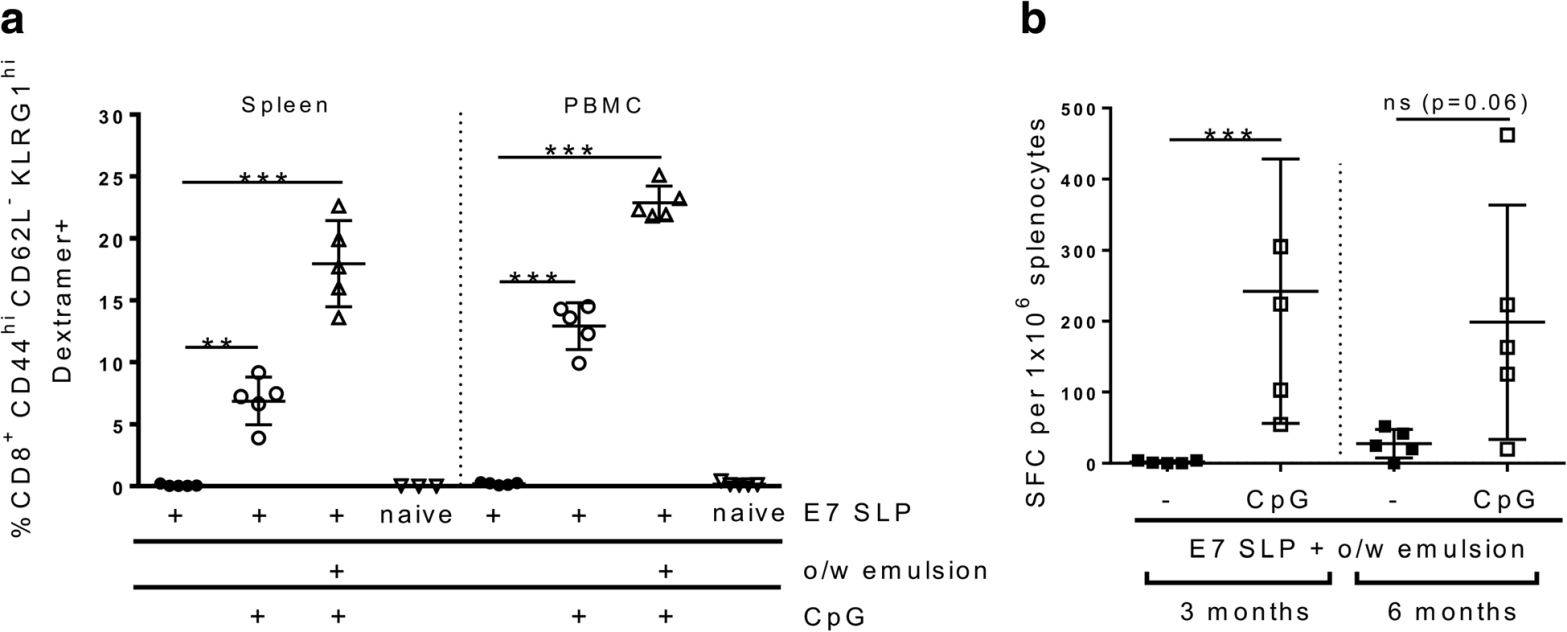
Adjuvanted E7 SLP promotes antigen specific memory CD8 T cells durable T cell functionality. Mice (n = 5) were immunized s.c. on day 0 and 21 with 20 μg E7 SLP + CpG +/− o/w emulsion. Splenocytes and PBMCs were isolated on day 28. **a** Cells were FACS analyzed for presence of E7 dextramer^+^ CD62L- CD44^hi^ KLRG1^hi^ populations. **b** Mice (n = 5) were immunized s.c. on day 0 and 21 with 3 μg E7 SLP + o/w emulsion +/− CpG. Splenocytes were isolated approximately 3 months (day 111) and 6 months (day 202) post second immunization. Cells were stimulated in vitro with E7 ME peptide and analyzed via ELISpot for IFN-γ secretion. Two-sided hypothesis test for comparisons using one-way ANOVA model; ns, not significant; **, *p* < 0.01; ***, *p* < 0.001

To assess the killing capacity of the antigen specific CD8 T cells induced by E7 SLP +/− CpG +/− o/w emulsion we performed an in vivo cytotoxicity study. Naïve splenocytes were loaded with E7 peptides or an irrelevant peptide (EBV gp350), mixed at a 1:1 ratio and transferred to vaccinated animals. Spleens from the mice that received the transferred cells were tracked for the E7 specific cell disappearance 4 h later (Fig. 4). Delivery of E7 SLP + CpG + o/w emulsion led to near 100% deletion of E7^+^ target cells, demonstrating higher activity than CpG or emulsion alone. We also observed the establishment of a substantial effector memory (CD62L^lo^ KLRG1^hi^) CD8 T cell population by 7 days post-second vaccination in both PBMCs and spleen (Fig. 5a). This population was E7-specific as it also stained positive for E7 dextramer. To determine the durability of this CD8 T cell population, we sampled E7-specific T cell responses at 3 and 6 months post second vaccination by ELISpot and found antigen-specific responses still detectable at those time points, which were substantially higher in SLP + CpG + o/w emulsion-immunized mice than in mice immunized with SLP + emulsion alone (Fig. 5b).

### SLP is superior to minimal epitope peptide for the induction of antigen specific CD8 T cells

**Fig. 6:**
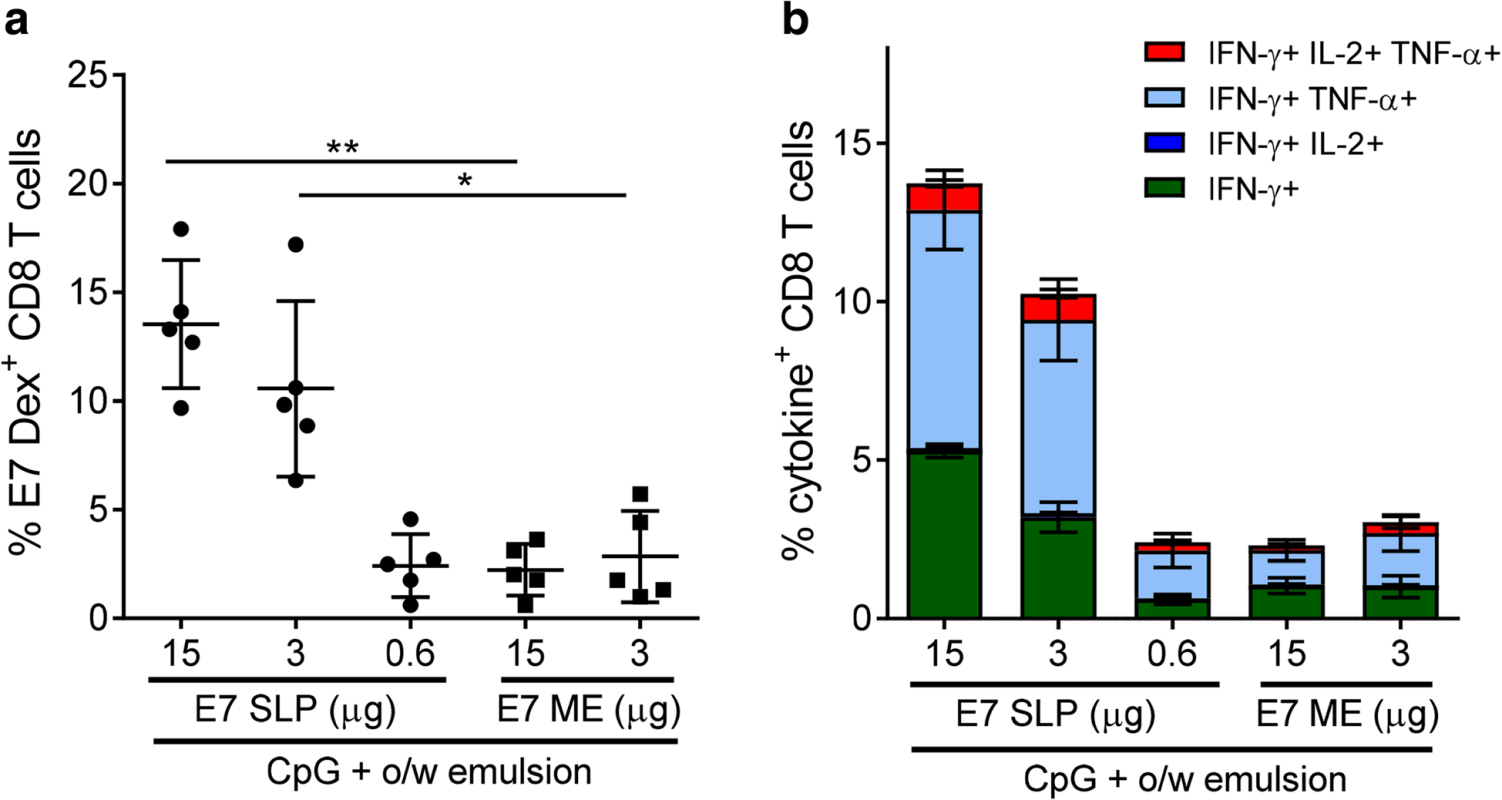
E7 SLP + CpG + o/w emulsion is optimal for robust CD8 T cell induction. Mice (n = 5) were immunized s.c. on day 0 and 21 with either 0.6, 3, or 15 μg E7 SLP or with 3 or 15 μg E7 ME peptide, in the presence of 20 μg CpG + o/w emulsion. Splenocytes were isolated on day 28. **a** E7 dextramer^+^ CD8 T cells were enumerated by FACS. **b** Cells were restimulated with E7 ME peptide for 5 h, then FACS analyzed for E7-specific multifunctional CD8 T cells intracellular expression of IFN-γ, TNF-α or IL-2. Two-sided hypothesis test for comparisons using one-way ANOVA model; *, *p* < 0.05; **, *p* < 0.01

Several studies have reported that utilizing SLPs that include flanking residues surrounding the epitope of interest as the antigenic component in vaccines offers superiority over vaccination with short peptides containing only the minimal CD8 determinant alone [17, 18]. To confirm that optimal CD8 T cell-inducing effects were achievable with SLP, we conducted a dose response in mice vaccinated with E7 SLP adjuvanted with CpG + o/w emulsion and compared to mice vaccinated with the same adjuvant formulations containing only the minimal epitope, RAHNIVTF. The SLP form of E7 was far more efficient at inducing the expansion of E7 dextramer-positive CD8 T cells (Fig. 6a) and E7-specific multifunctional CD8 T cells (Fig. 6b) than the RAHNIVTF minimal peptide, even though the RAHNIVTF H-2Db determinant was present at 4.8-fold molar excess in the short peptide formulation when compared to the E7 SLP formulation. Collectively, the data demonstrating high cytotoxic ability and durability of the CD8 T cell response generated with a E7 SLP + CpG + o/w emulsion vaccine supported the testing of this vaccine format in tumor-bearing mice.

### Vaccination with SLP + CpG + o/w emulsion provides robust protective effects in mouse TC-1 tumor model

**Fig. 7:**
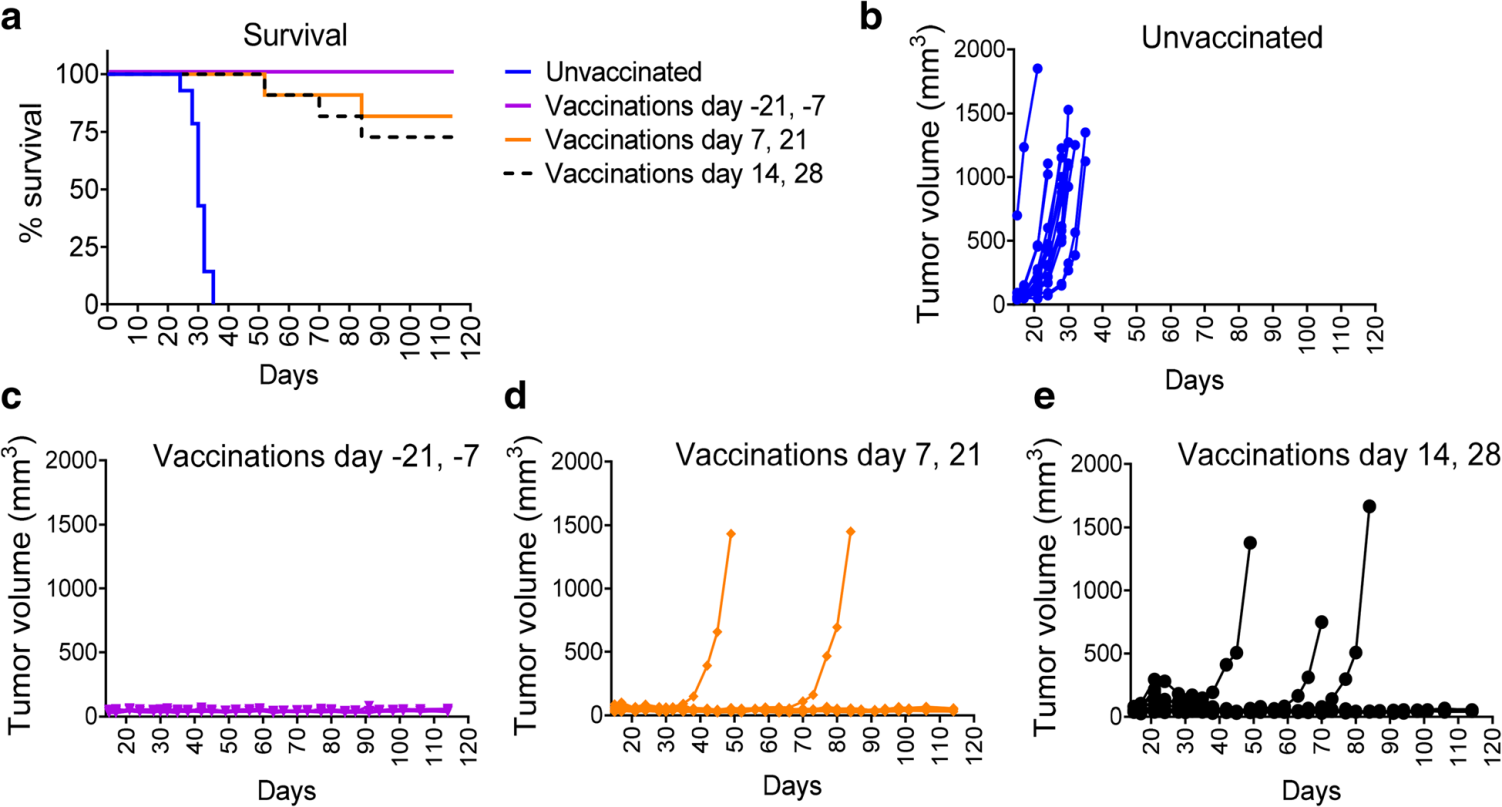
Profound TC-1 tumor growth inhibition induced by E7 SLP + CpG + o/w emulsion vaccination. Mice (*n* = 11 per group) were implanted s.c. (footpad) on day 0 with 2e4 TC-1 cells and then vaccinated s.c. (base of tail) with 20 μg E7 SLP + CpG + o/w emulsion according to 3 schedules: day − 21 and day − 7 (prophylactic); day 7 and 21 or day 14 and 28 (therapeutic). Mice were monitored for body weight and tumor volume 2-3X weekly until day 114. Mice with tumor size > 1000 mm^3^ were euthanized

The murine TC-1 syngeneic tumor model which expresses HPV16 E7 antigen was used to determine the potential for tumor growth inhibition induced after vaccination with SLP + CpG + o/w emulsion. TC-1 tumor-bearing mice were immunized under several schedules designed to inform on optimal dosing and timing. All groups received two immunizations two weeks apart. One group was treated prophylactically beginning twenty-one days prior to tumor implantation. The other groups underwent therapeutic vaccination beginning at either day 7 or day 14 post tumor implantation to determine how early vaccination was required to achieve optimal tumor regression. In unvaccinated mice, tumors became palpable at day 14–16 and grew rapidly to a size of 1000–1500 mm^3^ by day 24–28, when animals were euthanized according to ethical guidelines. Prophylactic vaccination protected 100% of the mice with no evidence for tumor growth through day 120 (Fig. 7). Therapeutic vaccination beginning at day 7 or day 14 post tumor implantation proved to be highly effective with no tumor growth in 9/11 (82%) and 8/11 (73%) of the mice, respectively. These results indicate the high potency of the SLP + CpG + o/w emulsion formulation and indicate that very strong protective responses can be achieved, even when vaccinating relatively late after tumor implantation.

**Fig. 8:**
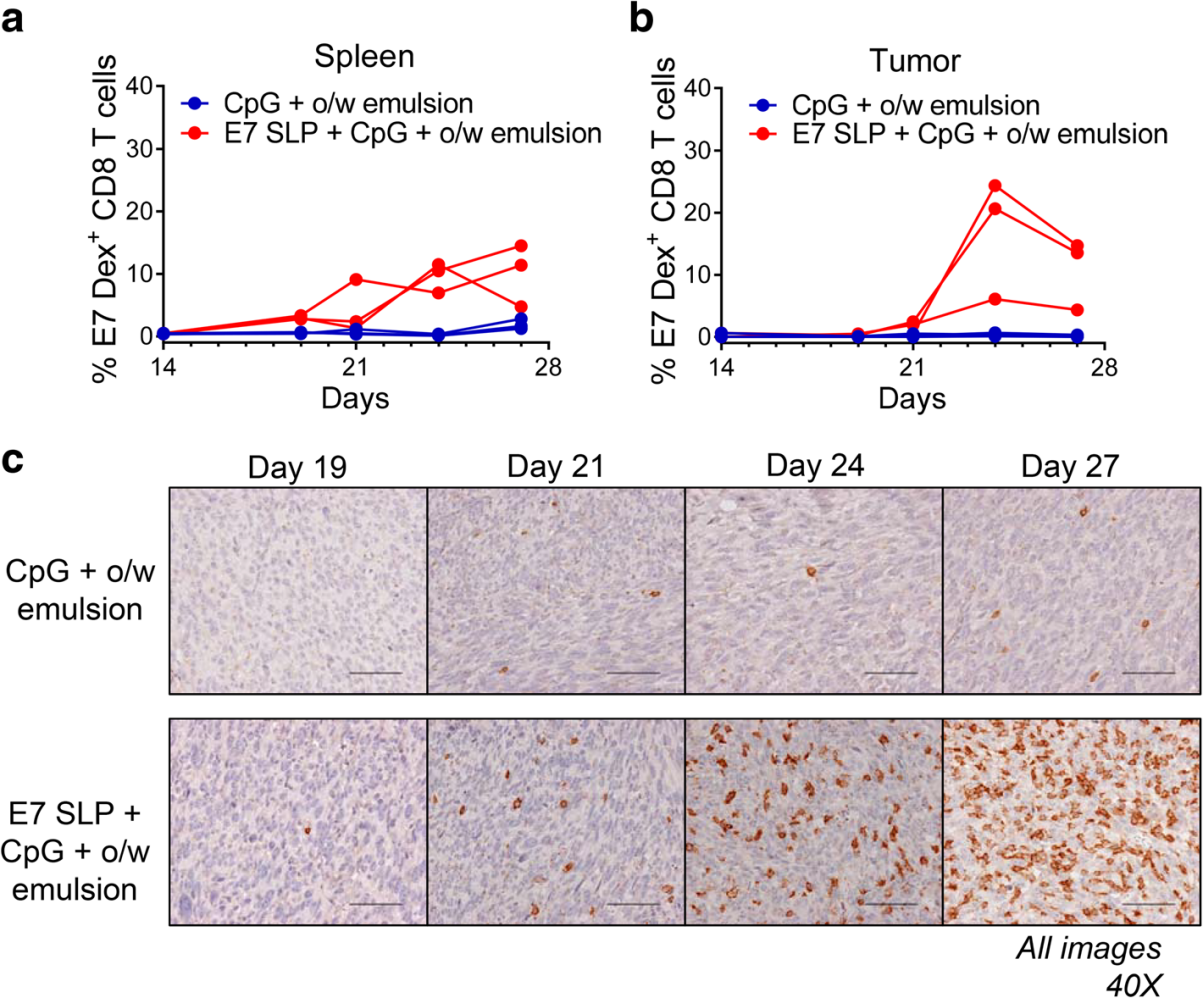
E7-specific CD8 T cells appearance in TC-1 mice vaccinated with adjuvanted E7 SLP. On day 0, mice (*n* = 20 per group) were implanted s.c. (footpad) with 2e4 TC-1 cells and then vaccinated s.c. (base of tail) on day 14 with 3 μg E7 SLP + CpG in w/o emulsion or adjuvant alone. Three mice from each group were sacrificed at 5 time points between days 14–28 and spleen and tumor tissue analyzed by FACS for E7-dextramer^+^ CD8 T cells within **a** total CD8^+^ population (spleen) and **b** total CD45^+^ population (tumor). **c** Tumor tissue from an additional mouse per group was immunohistochemically stained for the presence of CD8 T cells. All images 40X. Scale bars = 25 μm

To correlate tumor growth inhibition with antigen specific anti-tumor CD8 T cell activity, we immunized animals with CpG + o/w emulsion, to rule out formulation specific anti-tumor activity, or with SLP + CpG + o/w emulsion and tracked the appearance of E7 dextramer positive CD8 T cells in C57BL/6 mice implanted with TC-1. Mice were immunized on day 14 post tumor implantation when palpable tumor was apparent. Spleen and tumor tissues were analyzed for E7-specific CD8 T cells at the time points indicated in Fig. 8. E7 dextramer positive cells were detectable by day 19 in the spleen and day 21 in the tumor tissue and reached peak numbers around day 24 in the SLP + CpG + o/w emulsion group (Fig. 8a, b). Additionally, we detected a remarkable influx of CD8 tumor-infiltrating lymphocytes (TILs) in the central regions of tumors of SLP + CpG + o/w emulsion vaccinated mice by immunohistochemical staining, beginning at day 21 and peaking by day 27 (Fig. 8c). In the mice treated with the adjuvant only E7-specific CD8 T cell responses were not observed which indicates the requirement for exogenously delivered antigen for the priming of E7-specific CD8 T cells in this model system.

### Vaccinated mice are protected from re-challenge with TC-1

**Fig. 9:**
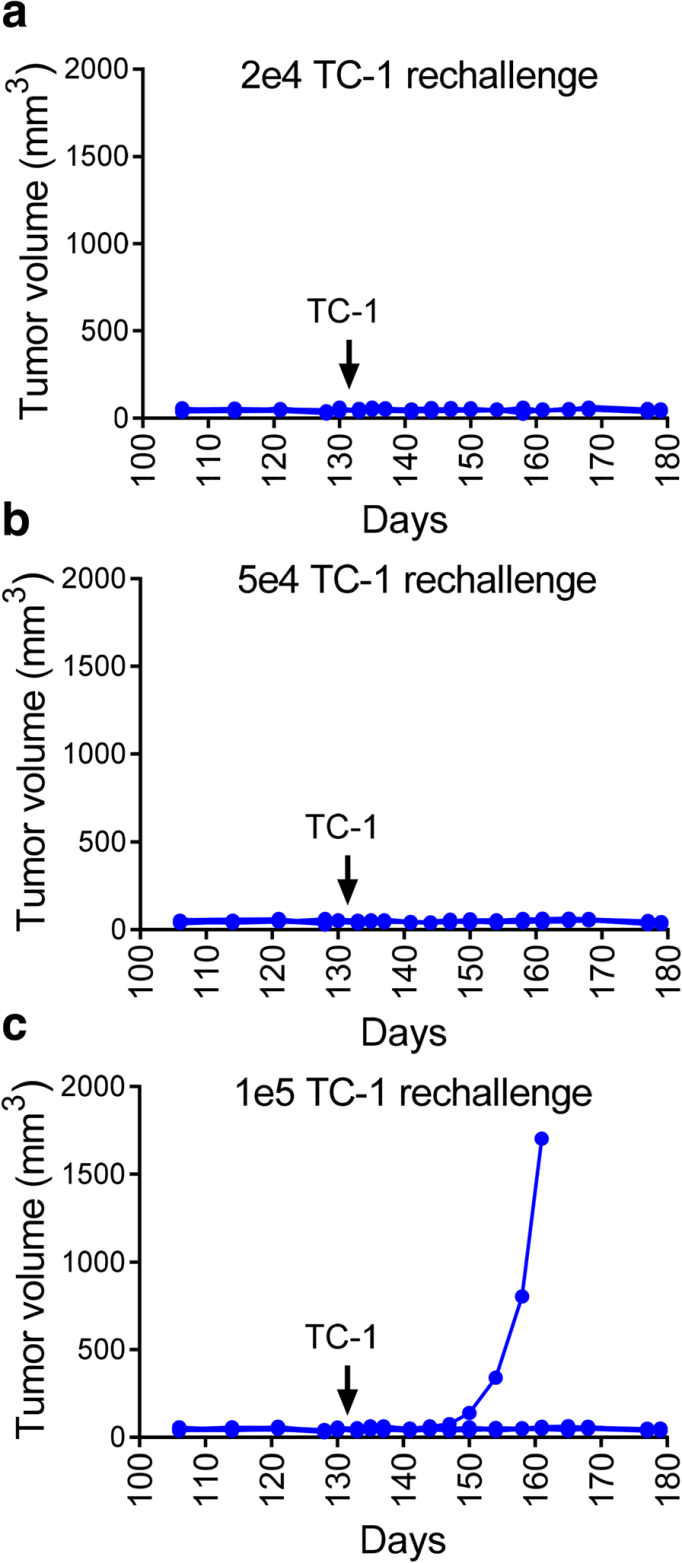
Vaccinated mice are protected from rechallenge with escalating doses of TC-1. 22 surviving mice from the study described in Fig. 6 were randomly distributed on day 120 into 3 groups and rechallenged s.c. (footpad) with of 2e4, 5e4, and 1e5 TC-1 cells. Mice were monitored for body weight and tumor volume 2-3X weekly until day 179. Mice with tumors size > 1000 mm^3^ were euthanized

To determine whether vaccinated mice experienced long-lasting protection, animals in which no tumor growth was observed were randomized on day 120 after initial tumor implantation and distributed into three new groups. These groups were re-challenged in the right footpad with 2e4 (same as day 0 challenge), 5e4, or 1e5 TC-1 cells (Fig. 9). All mice showed complete resistance to establishment of new tumor, with the exception of one mouse in the 1e5 group that showed tumor outgrowth and was sacrificed at day160. These results demonstrate that after therapeutic vaccination with E7 SLP + CpG + o/w emulsion, full protection can be achieved with up to a 5-fold higher secondary challenge dose of the TC-1 tumor cells.

## Discussion

Due to the low immune response generated against most peptide antigens, peptide-based therapeutic vaccines engineered to combat tumors and persistent viral infections must be adjuvanted to promote processing and class I presentation for consequent activation of robust CTL responses. We determined that pairing a tumor antigen peptide with CpG-C 2395 delivered in a squalene-based o/w emulsion was highly effective in promoting CD8 T cell responses in mice that were antigen-specific, long-lasting, multifunctional cytokine-expressing, and functionally cytolytic for target cells. This vaccine also proved particularly effective at conveying antitumor effects in the TC-1 tumor mouse model, greatly increasing survival, promoting tumor regression, and establishing a memory response that prevented tumor growth in animals re-challenged with higher doses of TC-1.

The efficacy of CpG-adjuvanted vaccines to treat established tumors in mice has been reported by other investigators. CpG ODNs or motifs have been combined in many different vaccine formats including: DNA plasmid encoding HPV16-E7 [19], bacteriophage-derived VLPs coupled to Melan-A/MART-1 peptide [20], lipoprotein-conjugated E7 antigen [21], and recombinant *Bordetella* adenylate cyclase bearing E7 polypeptide [22]. The presence of CpG has improved induction of cytolytic responses and/or tumor regression in each case. In a vaccine format more similar to that described herein, consisting of a 35-mer long peptide based on the HPV16 E7 RAHYNIVTF epitope formulated with CpG alone or with the w/o emulsion Montanide [23], investigators reported full tumor regression of TC-1 flank tumors after two s.c. opposite flank vaccinations at days 10 and 24. However, long-lasting protective responses were not fully evaluated as tumor growth was only recorded up to day 30. Since we observed tumor outgrowth occurring in some vaccinated mice post-day 30, it is unclear whether their vaccination regimen was truly curative. The authors also used 150 μg E7 SLP, while we have found that a much lower dose of 20 μg is very effective if delivered with an o/w squalene-based emulsion formulation, and that this formulation generates CD8 T cell responses of greatly increased magnitude when directly compared to those induced with Montanide.

W/o emulsions such as SEPPIC’s Montanide ISA51 and ISA720 have been used in multiple Phase I/II clinical trials and have shown some degree of efficacy, but also have shown safety signals at higher dose levels [24–26]. Furthermore, w/o emulsions like Montanide or IFA have been found to be suboptimal delivery systems for peptides in models of tumor vaccination as they are responsible for an antigen depot effect which causes CD8 T cells to home to the vaccination site rather than the tumor and to convert to an exhaustion phenotype [14]. On the other hand, pharmacology studies with o/w emulsions like MF59, the chemical composition of which the emulsion AddaVax used in our studies was based from, have demonstrated that the emulsion is rapidly drained away from the injection site and that the half-life of the antigen is not affected (reviewed in [27]). MF59 has been shown to induce strong CD4 T cell responses and has been incorporated into some flu vaccines [28] and recently o/w emulsions have been shown in animal models to generate strong cytotoxic CD8 T cell responses when paired with CpG against viral antigens [29].

Combining TLR agonists with an emulsion delivery vehicle is thought to increase the stability of the agonist as well as to improve antigen uptake and activation of APCs. The improved activity of TLR agonists by incorporation into a squalene-based o/w emulsion formulation has been demonstrated unequivocally with TLR4L. Prominent among this class of adjuvants is GLA-SE, in which the synthetic TLR4 agonist glucopyranosyl lipid A (GLA) is incorporated during the microfluidization process into the resultant squalene-based stable emulsion (SE) [30]. GLA-SE demonstrates clear superiority over GLA delivered either with aluminum salts or in aqueous-soluble micelles [31, 32]. A more limited data set has indicated that CpG combined with emulsion may also show enhanced efficacy [33, 34]. In line with these findings, we found that the equivalent dose of CpG without emulsion is less efficient in the induction of CD8 T cell responses than CpG + o/w emulsion, providing further support for the use of this adjuvant combination. A recent report by Song et al. demonstrated the improved efficacy of CpG when delivered by the novel w/o squalene emulsion PELC [35]. E7 peptide delivered by CpG/PELC resulted in higher E7-specific CD8 T cell induction, in vivo cytolytic activity, and protection in a TC-1 model through day 50, compared to peptide plus either adjuvant components alone [35]. The formulation used in the study was the H-2Db minimal determinant of E7 rather than the SLP, which likely accounts for why we observed higher magnitude CD8 T cell IFN-γ expression, higher cytolytic function, and full protection through day 130 in our study as we demonstrate that the increased ability of SLP to drive CD8 T cell responses when compared to minimal peptides.

## Conclusions

Peptide vaccines targeting cancer have not achieved the desired clinical endpoints of robust and broad CTL effects and vigorous antineoplastic activity [12, 13]. Much of this lack of success may be due to suboptimal adjuvant formulations (e.g. Montanide, GM-CSF) and antigen formats that cannot initiate the cross-presentation process in DCs that is vital for the promotion of robust CTL responses critical for antitumor immunity [14]. Peptide vaccines with TLR9-based adjuvants have already demonstrated CD8 T cell-inducing ability in the clinic [8, 36]. The next steps in the evaluation of the SLP + CpG + o/w emulsion should include and evaluation in non-human primates before study in humans. Our data support the further development and optimization of adjuvant formulations that demonstrate a pronounced ability to promote CD8 T cell responses for antitumor applications and as potential components of combination therapy with checkpoint inhibitor agents such as anti-PD-L1.

## Additional file

Additional file 1: Flow cytometry data presented in tabular form. (XLSX 48 kb)

## Abbreviations

CpG-C: Cytosine phosphate Guanine type C
CTL: cytotoxic T lymphocyte
FACS: fluorescence-activated cell sorting
HPV: human papilloma virus
ICS: intracellular cytokine staining
O/W: oil-in-water
ODN: oligodeoxynucleotide
PD-L1: programmed cell death-Ligand 1
SLP: synthetic long peptide
T_EM_: T effector memory cell
TLR: toll-like receptor
W/O: water-in-oil
